# Views from the global south: exploring how student volunteers from the global north can achieve sustainable impact in global health

**DOI:** 10.1186/1744-8603-9-32

**Published:** 2013-07-26

**Authors:** Brian DO Ouma, Helen Dimaras

**Affiliations:** 1 Daisy’s Eye Cancer Fund-Kenya, Nairobi, Kenya; 2 The Department of Ophthalmology and Vision Science, University of Toronto, Toronto, Ontario, Canada; 3 The Division of Visual Science, Toronto Western Research Institute, Toronto, Ontario, Canada; 4 The Hospital for Sick Children, 555 University Ave, Room 7260, Toronto, Ontario M5G 1X8, Canada; 5 Daisy’s Eye Cancer Fund-Canada, Toronto, Ontario, Canada

**Keywords:** Global health, Study abroad, Student volunteers, Volunteer tourism, Critical engagement, Medical education

## Abstract

**Background:**

The body of research and practice regarding student volunteer abroad experiences largely focuses on ensuring the optimal learning experience for the student from the Global North, without equivalent attention to the benefits, if any, to the host institution in the Global South. In this debate article, we examine an often overlooked component of global student volunteer programs: the views of the local partner on what makes for a mutually beneficial partnership between volunteers from the Global North and institutions in the Global South.

**Discussion:**

To guide our discussion, we drew upon the experiences of a Kenyan NGO with a Canadian student volunteer in the summer of 2012, organized via a formalized partnership with a Canadian university. We found that the approach of the NGO to hosting the student mirrored the organizational behaviour theories of Margaret J. Wheatley, who emphasized a disorderly or ‘chaotic’ approach to acquiring impactful change, coupled with a focus on building solid human relationships. Rather than following a set of rigid goals or tasks, the student was encouraged to critically engage and participate in all aspects of the culture of the organization and country, to naturally discover an area where his priorities aligned with the needs of the NGO. Solid networks and interpersonal connections resulted in a process useful for the organization long after the student’s short-term placement ended.

**Summary:**

Our discussion reveals key features of successful academic volunteer abroad placements: equal partnership in the design phase between organizations in the Global North and Global South; the absence of rigid structures or preplanned tasks during the student’s placement; participatory observation and critical engagement of the student volunteer; and a willingness of the partners to measure impact by the resultant process instead of tangible outcomes.

## Background

In the last decade we have experienced a boom in the number of global health volunteer and study abroad opportunities in the Global South specifically targeted to students from the Global North; we use the terms ‘Global North’ and ‘Global South’ to broadly refer to a socio-economic divide rather than a strictly geographical one. With experiences ranging from international medical electives, internships and semesters abroad with integrated service-learning components, to ‘voluntourism’ programs arranged by tour companies for a fee, the benefits to the student participating in such programs are widely lauded: they acquire intercultural competencies [1-4], undergo personal growth [5,6] and feel their academic experience is enriched upon their return home [7-9].

Interestingly, most available literature on volunteer and study abroad programs is focused on determining the benefits to the students [2-6,8,10-16] or on how to plan the course or program to provide the optimal learning experience [17,18]. In contrast, relatively little attention has been paid to the impact of such programs on local institutions that host volunteers from the Global North, for which the limited research shows without appropriate attention, negative consequences could ensue [19,20]. To be sure, several criticisms abound, though not always published: examples of students abroad who undertake roles without appropriate skill set or training; others who display predominantly a vacation mindset [21]; also the idea that volunteers spend too little time with the host to add value or have any lasting impact, and instead create more work for already overstressed organizations.

The argument has been presented that volunteer abroad programs perpetuate an overly simplified view of global development, as well as the flawed notion that more young, unskilled labour is the way out of the development conundrum [22]. Clearly, care must be taken in the design of such programs to avoid these negative and often unethical consequences. This paper will focus on ways in which existing programs, specifically those that are embedded within formal academic institutions in the Global North, can ensure a mutually beneficial partnership with their partners in the Global South, as a small step in achieving the broader goals of global health and development.

In this debate article, we offer insights from the experiences of a Kenyan NGO with a Canadian student volunteer in the summer of 2012, revealing an often overlooked and under-reported component of global student volunteer programs: the views of the local partner on what makes for a mutually beneficial partnership between volunteers from the Global North and institutions in the Global South. We suggest that unstructured approaches to global student volunteer programs, which emphasize relationship building over programmatic outcomes, provide a greater possibility of developing mutually beneficial partnerships between the Global North and South that will meet global health challenges over the long-term.

## Discussion

### The context

#### Setting up the partnership – mutual respect and responsibility

Daisy’s Eye Cancer Fund (DECF, http://www.daisyfund.org) is a small Kenyan NGO with the mission to save the life and sight of children afflicted with the rare eye cancer retinoblastoma. Based in Nairobi, DECF runs on a small operating budget and employs a staff of three, relying heavily on the assistance of multiple volunteers, mostly local. The opportunity to partner with an academic institution in the Global North was welcomed as a way in which to expand the capabilities and reach of the organization on the ground in Kenya, and strengthen ties with strong international partners.

The University of Toronto Center for International Experience (UofT CIE) aims to partner with small NGOs in the Global South, with the goal of sending strong students to participate in a ‘service learning project’ (learning by volunteering) related to their course of study. The students are responsible for identifying a UofT faculty member to guide their academic development, and are required to submit a research paper for academic credit.

A UofT faculty member with ties to DECF (HD) connected the two institutions, and a joint grant application was developed and submitted to obtain funding to cover the costs of hosting student volunteers. Established research connections between the NGO and three UofT faculty supported the potential for sustained, long-term impact beyond the relatively short tenure of the individual student abroad placements. An agreement between DECF and UofT CIE outlined the formal relationship and responsibilities of each party. Each partner viewed the study abroad program as a tool to nurture sustainability and strengthen local capacity in health. This mutual purpose is an essential component of study abroad programs so reduce the ethical problems that may undermine its success [23].

#### Selection of the student – a mutual decision

After obtaining grant funding, UofT undergraduate students from any discipline were invited to apply for a summer volunteer placement, recognizing that global health is a multi- and trans-disciplinary field [24]. Placements are highly competitive and typically the chosen students have excellent academic standing, strong personal character and a history of extra-curricular and/or community involvement. The thorough selection process underpins the point that study abroad placements are a privilege to be earned [23], and may encourage students to be more respectful and conscientious while abroad.

A student of economics was selected by UofT CIE to work with DECF based on evaluation of a statement of interest, CV, and academic performance. The student’s experience and training in economics, as well as a keen interest in exploring the role of microfinance in development, appeared to complement the burgeoning microfinance initiatives of DECF that assist mothers of cancer patients with payment of National Health Insurance Fund dues to cover cancer treatment. DECF vetted the student’s application package and accepted the student. From June 1 to September 2, 2012, DECF hosted the undergraduate student in Nairobi. The student arranged for an academic supervisor at UofT to guide his academic development, with the requirement to submit a paper on microfinance for UofT credit upon completion of the placement abroad. The student’s precise tasks for the service learning component within the DECF team was not determined prior to his arrival to Kenya, nor was this a requirement of the academic program. During the internship, there was regular online communication between Kenyan and Canadian NGO offices via Skype to keep on top of the student’s progress.

#### Program design - a mutual purpose and benefit

Integral to our program design was the early establishment of an equal partnership between parties, with a mutual accountability to each other and their funder. This approach is not very well documented in the literature about academic student abroad programs. For example, a study from a program in rural Haiti is entirely concerned with developing the students of the Global North in terms of their surgical training, cultural competencies and learning to work in low cost settings, with no mention of the relationship with or accountability to the local partner [25]. Similar research which reported the effects of international medical electives on American physician career choice without reference to local partner experiences [15], prompted commentary warning that the enthusiasm for ensuring optimal student experiences may lead to the disregard of potential ethical challenges with negative consequences for the local partner of host [26].

We argue that the missing factor in most academic study abroad programs is feedback from local partners, which should be embedded in the program design. Along these lines, a study in Guatemala aimed to elucidate how short-term medical volunteers were perceived by various stakeholders [20]. It was shown that the presence of American physician volunteers appeared to undermine the credibility of local physicians, albeit being just as qualified to practice [20]. While stakeholders perceived an improved access to healthcare brought about by such initiatives, they recommended increased ‘coordination with and respect for Guatemalan health care providers’ as a major point for improvement [20]. Had there been such coordination to design the program with all stakeholders in the first place, the respect the key stakeholders were seeking might have naturally developed.

We propose a shift in the design of academic volunteer abroad programs to formally incorporate a mutual benefit design at their inception, with equal input from all stakeholders. The experiences from each stage of the program could be incorporated to the program design for the next crop of students, such that the program is ever-evolving and adapts to the needs of all parties involved.

### Global south viewpoint: 3 essential components for sustainable outcomes of student volunteer programs

#### 1. Use an unstructured approach

We argue that since most volunteer abroad programs have focused on how to get the best experience for the student, they often lack a focus on the mutual benefit. It is no wonder then, that many students have a set of priorities and goals for their volunteer abroad opportunity before they even step foot outside the arrivals terminal at the airport. These goals might be related to the requirements of the partner in the Global North (e.g. conditions for academic credit), which may or may not be beneficial to the partner in the Global South.

DECF, like many other small, relatively new organizations, does not operate on strict structures, as it is still growing and is in a constant state of change. Employees and volunteers operate with a high level of autonomy, trust being a key factor in the workplace. The volunteer student arrived at a time of restructuring within the NGO, and there was no specific job for him on the ground other than the vague goal of participating in a pre-existing microfinance project. Prior to the student’s departure from Canada, he was briefed to make him aware of the unstructured nature of his volunteer placement, and to emphasize that he was to get to know the people and local context as best he could before launching into his independent study.

The decision to keep the description of the student’s role vague within the NGO was deliberate. This method may be frustrating for students from the Global North, who might be used to a goal-oriented work culture. However, when students are particularly very bright and motivated, as those selected for such programs often are, their ideas may be beyond the capabilities of a stressed and poorly-resourced organization, and without appropriate experience within the right context, likely misplaced and not relevant to the local situation. Continuing on a path aimed at meeting preconceived goals rather than one aimed at creating mutually beneficial goals, will likely end in failure. Regarding the absence of a clearly mapped out role, the student remarked:

*“It was tough figuring out something when I didn’t know what the right direction was and how it would all come together*. *I just kept believing that something would come*, *and it did*: *like a jigsaw puzzle where you don*’*t know at first where all the pieces will fit*.”

With a rough direction to focus on microfinance, the student naturally found his preferred niche and explored the role of microfinance on poverty alleviation as it related to the experiences of families dealing with childhood cancer. He evaluated the existing DECF program alongside his own directed reading of published microfinance programs, and modeled the potential impact of introducing a microfranchising model instead.

The unstructured approach of DECF brings to mind the theories on leadership and organizational behaviour of Margaret J. Wheatley [27-29], who emphasized a disorderly or ‘chaotic’ approach to acquiring impactful change, coupled with a focus on building solid human relationships, rather than following a set of rigid goals or tasks. Indeed, Wheatley’s theories have previously been applied in the planning stages of a nursing medical elective program in Nepal, where authors reported a successful program, albeit from the perspective of the academic institution in the Global North [30].

We propose that some degree of flexibility is required at the outset, and a conscious effort to remain without structures to avoid being locked into goals that may be artificial. This allows an open path for student and local host to create something of value that is mutually beneficial.

#### 2. Encourage participatory observation and critical engagement

Without a defined role, DECF staff encouraged the student to immerse himself within the organization, observing all roles as they fit within the NGO vision and mission, and to see and experience firsthand challenges and opportunities. Inherent in this process the student formed solid bonds, developed mutual trust, and built strong relationships with many stakeholders – everyone from the NGO employees to members of their broader network in areas such as government, healthcare, and finance.

In Kenya, strong interpersonal relationships are at the core of daily life, including at the workplace. It has been suggested that fostering good relationships within organizations helps unleash hidden creative power; preplanned strategies, on the other hand, may impede the cultivation of such relationships [27]. In the field global health, where progress demands innovation, surely it is creativity that we are after?

Encouraging the student to totally immerse himself in the organizational culture, as well as the culture of the country and its people, set the stage for him to naturally discover what role he and his ideas might play within the broader picture DECF and its programs. With this knowledge and lack of structure, the student was empowered. With his free, unstructured time, he expressed that he felt he owed that time to the organization. Essentially, the student engaged every person he met, every new connection, and thought back to how it might be useful to DECF, how it might shed light on the challenges or how he might learn about the business and social culture of Kenya. Specifically, he interviewed local leaders in banking and microfinance, as well as NGO workers from Nairobi and surrounding regions to gain insight into local organizational policies and procedures. In addition, he gained an intimate understanding of and respect for local people and practices, stating:

“*One thing that I had to keep fighting was my Western tendency to think that* ‘*our*’ *way is better*, *and also to only question and analyze what was happening*, *and not criticize*. *Meeting new friends there and spending time with them socially also enriched my ability to understand things and how to interact personally with Kenyans*. *They showed me places and introduced me to social circles outside that of the NGO*. *Also*, *I found having the chance to interact with DECF staff personally*, *learning about them and their past*, *enriched my understanding of peoples paths in life* (*in Kenya*), *coming from different households and regions*.”

This critical engagement was essential for the student’s process of ‘self-organization’ [27], to naturally determine his role within the organization. ‘Limited critical engagement’ is one major critique of the gap year abroad – that is, observation in the absence of experience, tinted by preconceived notions of life in a developing country [22]. This type of biased observation runs the risk of turning into a false sense of authority upon return to the Global North [22], and adding to the growing misconceptions about global development.

Participatory observation, on the other hand, gives a truer picture of the situation, and is also cited as having a better psychosocial effect on student, discouraging a vacation mindset and allowing the student to truly belong [31]. Out of this sense of belonging and experienced understanding of the host organization, the student is more likely to align their priorities with the NGO and self-organize into a role that can create something of value to each partner [27]. The challenge to keep the needs of the student volunteer from averting human or material resources from the overtaxed healthcare systems they are trying to assist is very real [32].The added benefits of our approach to a small organization are that the student operates autonomously and out of internal motivation, and is less likely to result in lost productivity of busy NGO employees catering to the needs of a lost or confused student.

#### 3. Measure impact by the journey, not the destination

True sustainable change takes time to develop, and the reality is that most student volunteer programs run for 6 months or less, making this very difficult to achieve. For DECF, the impact of the student volunteer was measured in terms of the process that was initiated, and not by finite, tangible outcomes. The unstructured approach that encouraged the student to observe and engage to find his niche, initiated the phenomenon of emergence, the natural precursor to change [27]. Though his academic work remained focused on writing a critique of the impact of microfinance on poverty alleviation, his role within DECF emerged into one of working with the organization to develop and document processes regarding internal communication, reporting and accountability. The networks formed by the student created something of value for the organization from which to further develop their programs and practices. Furthermore, the relationships between the NGO staff and the student enhanced the local NGO’s understanding of their academic partner’s motivations and interest in the partnership. This is another building block to a long-standing partnership between organizations, which starts with strong interpersonal relationships. Though difficult to measure by conventional means, the emergent process has the potential for powerful, meaningful change.

### The way forward

We note that global health student volunteer programs geared towards students from the Global South studying in the Global North are few and far between. This simple observation may undermine the development of truly mutually beneficial programs, as it reinforces the flawed notion that the assistance of the Global North is required for developing health systems in the Global South. More programs like the McMaster University’s International Pediatric Emergency Medicine Elective, which brings together medical students from Canada and the Middle East to study together in Canada [33], may start to turn the tide towards collaborative approaches to global health education. Health as a part of the global commons emerges as a significant concept when partnerships are approached following the principles of mutual respect and sharing.

The recent interest in exploring ‘reverse innovation’ [34] for global health [35] (i.e. the application of innovations from the Global South to the health challenges of the Global North) has promise to promote bidirectional partnerships (even though the use of the term ‘reverse’ itself erroneously implies that the natural course of innovation goes from more-developed to less-developed countries). Nevertheless, this new interest could result in partnerships that truly attempt to be bidirectional and mutually beneficial, with the core principles highlighted by our case being an important part of their development.

### Limitations

Our case study of global student volunteerism reveals three salient principles to consider in the design of North–south partnerships for global health. Though successful in our experience, we by no means suggest that our single experience is representative of the broader group of student volunteer programs in existence, though it does stimulate discussion in the field. Our approach may yield different results in other settings and contexts. DECF has a small staff and receives 1–2 students at a time, so organizations with a higher influx of students may find an unstructured approach difficult to manage or not suitable to their needs. For international medical electives, a formal curriculum [36] with consistent supervision [37], rather than an unstructured approach, may meet academic goals more clearly. More extensive pre-departure training for students to develop global health competencies may complement both unstructured and structured approaches to study [38,39]. Overall, the goals to attain a mutual purpose, build bilateral relationships and incorporate the often-forgotten views of the local partner remains highly relevant no matter which approach is taken. We offer our experience and perspective mainly to stimulate discussion and experimentation regarding student abroad program design.

### Summary

Our discussion reveals key principles that may enhance success of global health student abroad experiences: equal partnership in the design phase between organizations in the Global North and Global South; the absence of rigid structures or preplanned tasks during the student’s placement; participatory observation and critical engagement for the student; and a willingness of the partners to measure impact by the resultant process, not the outcome. Further experimentation with student volunteer program design will build theory and experience in this field.

## Abbreviations

DECF: Daisy’s eye cancer fund; UofT: University of Toronto; CIE: Center for international experience; NGO: Non-governmental organization

## Competing interests

The authors declare no competing interests.

## Authors’ contributions

BO and HD conceptualized the general framework of the article, based on their experiences during the planning and implementation of the DECF-UofT CIE volunteer abroad program. BO contributed to data collection and manuscript editing. HD collected the relevant data, and drafted and edited the manuscript. Both authors read and approved the final manuscript.

## Authors’ information

BO: CEO, Daisy’s Eye Cancer Fund Kenya. HD: Assistant Professor, University of Toronto; Affiliate Scientist, Toronto Western Research Institute; Adjunct Scientist, SickKids Research Institute.
